# Monitoring Site-Specific Fermentation Outcomes via Oxidation Reduction Potential and UV-Vis Spectroscopy to Characterize “Hidden” Parameters of Pinot Noir Wine Fermentations

**DOI:** 10.3390/molecules26164748

**Published:** 2021-08-05

**Authors:** Gordon A. Walker, James Nelson, Thomas Halligan, Maisa M. M. Lima, Andre Knoesen, Ron C. Runnebaum

**Affiliations:** 1 Department of Viticulture & Enology, University of California, Davis, CA 95616, USA; gawalker@ucdavis.edu (G.A.W.); mmlima@ucdavis.edu (M.M.M.L.); 2 Department of Electrical and Computer Engineering, University of California, Davis, CA 95616, USA; jjnel@ucdavis.edu (J.N.); aknoesen@ucdavis.edu (A.K.); 3 Department of Chemical Engineering, University of California, Davis, CA 95616, USA; twhalligan@ucdavis.edu

**Keywords:** oxidation reduction potential, Pinot noir, fermentation, yeast metabolism, process control

## Abstract

Real-time process metrics are standard for the majority of fermentation-based industries but have not been widely adopted by the wine industry. In this study, replicate fermentations were conducted with temperature as the main process parameter and assessed via in-line Oxidation Reduction Potential (ORP) probes and at-line profiling of phenolics compounds by UV-Vis spectroscopy. The California and Oregon vineyards used in this study displayed consistent vinification outcomes over five vintages and are representative of sites producing faster- and slower-fermenting musts. The selected sites have been previously characterized by fermentation kinetics, elemental profile, phenolics, and sensory analysis. ORP probes were integrated into individual fermentors to record how ORP changed throughout the fermentation process. The ORP profiles generally followed expected trends with deviations revealing previously undetectable process differences between sites and replicates. Site-specific differences were also observed in phenolic and anthocyanin extraction. Elemental composition was also analyzed for each vineyard, revealing distinctive profiles that correlated with the fermentation kinetics and may influence the redox status of these wines. The rapid ORP responses observed related to winemaking decisions and yeast activity suggest ORP is a useful process parameter that should be tracked in addition to Brix, temperature, and phenolics extraction for monitoring fermentations.

## 1. Introduction

Wine fermentations are typically monitored using Brix (density measurement correlated with sugar percentage) and temperature. Generally, musts start out between ~22–26 Brix and decrease until the fermentation reaches a negative Brix value, which is due to the conversion of sugars into less dense ethanol. The rate of change of Brix is dependent on a number of factors, including grape variety, must microbiome, inoculation, yeast strain, nutrients (macro and micro), oxygen availability, temperature, and ethanol concentration [1,2,3]. During the course of fermentation, the temperature generally starts low, increases due to yeast metabolism (often requiring temperature control to mitigate the deleterious effects of excess heat), and decreases in late fermentation, when yeast is less active [4]. Controlling temperature offers one approach to change rates of fermentation by modulating yeast metabolism. Additionally, the extraction of phenolics compounds, including anthocyanins, is a function of temperature [5]. While Brix is generally sufficient to follow fermentation progress and temperature can be used to control activity, measured changes in these parameters are generally not collected in real-time; problematic fermentations, therefore, often do not become apparent until sugar consumption by yeast has slowed or become “stuck” [6]. Thus, process parameters for winemaking that provide insight into yeast metabolism and the changing chemical fermentation environment in real-time will be of considerable value to the industry.

Redox potential, or Oxidation Reduction Potential (ORP), is a process parameter currently utilized for biofuels production, wastewater treatment, dairy processing, and food safety determination [7,8,9,10,11]. The redox potential of a solution (extracellular) determines the thermodynamically stable forms (including oxidation state) of species in solution, each of which can facilitate reactions at varying rates [12]. Thus, ORP determines the favorability of relevant half-reactions to occur [13]. A relevant half-reaction in wine-like conditions is the conversion of Fe(II) to Fe(III), which is dependent on tartaric acid as a ligand, along with pH and temperature in the presence of oxygen [14]. The measurement mechanism of ORP is electrochemical (similar to pH) but is a composite measurement that is influenced by pH, dissolved oxygen (DO), temperature, and chemical half-reactions taking place in solution [15]. This pseudo steady-state measurement is fundamentally different from non-steady-state work that has been performed with cyclic voltammetry methods, which use a flow of electrons into or out of the system [16,17]. The redox conditions play an important role in dictating chemical reactions during wine aging, as well as during fermentation.

While redox potential is understood and employed in a variety of other anaerobic processes, minimal work has been conducted on characterizing wine fermentations [18,19,20]. Previous work has demonstrated the effectiveness of using ORP as a strategy for controlling the activity of biological systems [15]. In low-oxygen yeast fermentations, changes in redox potential are primarily driven by the physiological and metabolic status of the yeast population: as yeast depletes molecular O_2_, produces CO_2_, and excretes reductive metabolites, it causes the ORP to decrease [21]. As fermentation progresses, an “ORP minimum” is reached, which corresponds with an increase in hydrogen sulfide (H_2_S) due to yeast amino acid metabolism and spontaneous extracellular reduction of elemental sulfur at low redox potential [18,20,22]. The ORP minimum value correlates with the transition from exponential to stationary phase as mediated by H_2_S quorum sensing in the yeast population [23]. The goal of this work is to build on the understanding of how ORP changes with initial must conditions, fermentations progression, and relates to phenolic extraction and elemental profiles across different vineyard sites. It is expected that the combination of these data, typically not used in wine fermentations, will help to elucidate previously “hidden” contributions of vineyard sites to fermentation outcomes and wine chemistry.

Another important metric for monitoring fermentations is the extraction of phenolics compounds. Extraction is a crucial part of the winemaking process, important for wine quality and organoleptic perception [24,25]. The concentration of pigments in the grapes is a reflection of the berry physiology and combination of environmental factors [26,27,28]. The Adams–Harbertson assay was developed to quantify phenolic content, enabling correlations to sensory properties such as color compounds (e.g., anthocyanins) and astringency perception (e.g., tannins) [29]. However, due to limitations of time and trained labor, a faster, reliable way to predict important phenolics fractions extracted into wine was required. UV-Vis spectral analysis has demonstrated robust correlations with Adams–Harbertson assay and provides an opportunity for a more rapid evaluation (nearly real-time) of these important winemaking parameters [30]. To help elucidate the site-specific characteristics and understand the kinetics of phenolics extraction, particularly as it applies to the identical clone of a variety grown on different sites, UV-Vis spectrometry was used throughout primary fermentation to assess extraction of phenolics and anthocyanins [31].

To understand site-to-site variation, the elemental composition of wines has been analyzed to “fingerprint” or assess wine provenance from a particular geographic area [32]. Previous work has demonstrated the importance of elemental profiles for determining site-specificity but also revealing insights into the soil composition, farming practices, weather patterns, and inherent biological activity associated with particular elements [33]. Yeast fermentation performance can be strongly influenced by the concentration of particular elements [1,34]. The concentration and composition of elements (Fe and Cu especially) influence the redox potential of a must/wine, can impact yeast metabolism during fermentation, and play a key role in wine aging [35,36]. Quantifying the elemental profile of vineyard sites is anticipated to correlate with site-specific factors that influence fermentation outcomes.

**Figure 1:**
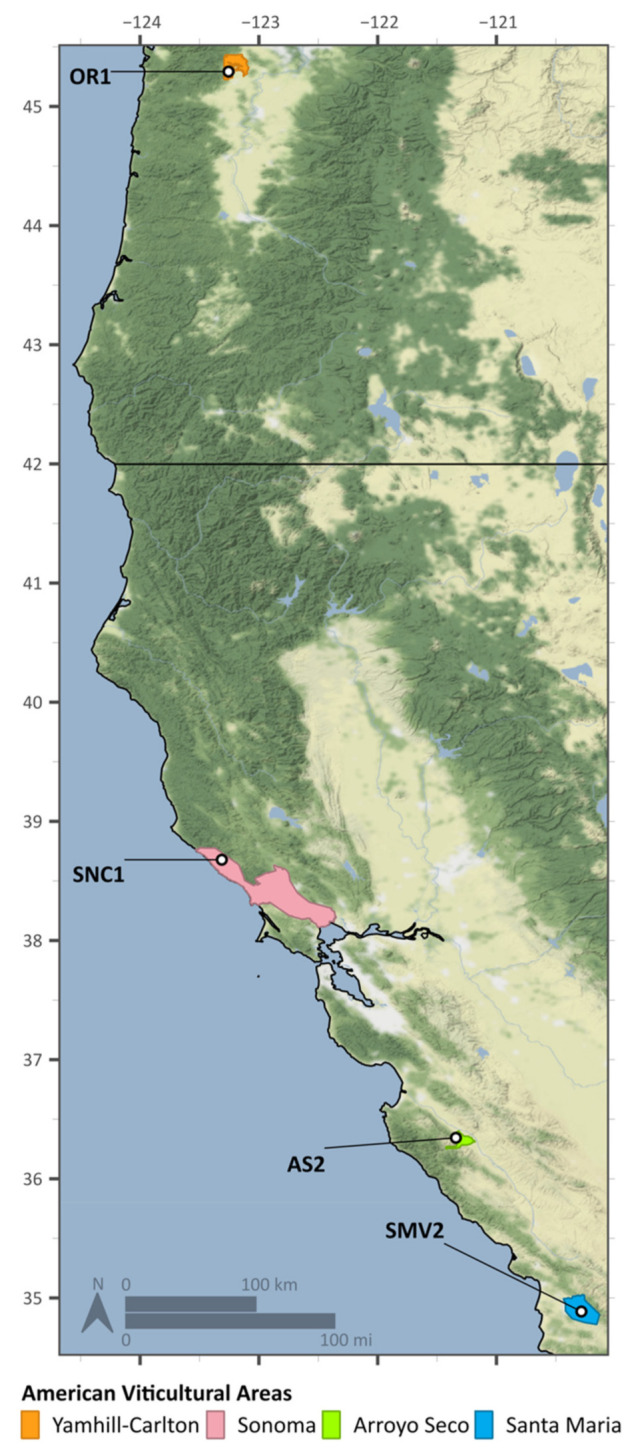
Geographical spread of vineyard sites included in this study. Sites range from the central coast of California up to Oregon. The American Vineyard Areas (AVAs) represented are: SMV2—Santa Maria Valley, AS2—Arroyo Seco, SNC1—Sonoma (Coast), and OR1—Yamhill–Carlton (Willamette Valley).

The provenance of grapes has been demonstrated to result in a discernable (and quantitative) effect on the finished wine [37]. However, the variables of site-specificity are difficult to define because there are numerous biotic and abiotic factors that contribute to the characteristics of a wine [38,39]. The final wine product is an intersection and interaction of geographic and environmental factors, along with all the process decisions undertaken [2,4]. For this work, a subset of four vineyard sites was selected that displays consistent fermentation kinetics from year to year: two sites each producing fruit that results in “faster” and “slower” fermentations. The vineyards selected represent sites spanning from the central and northern coast of California up to Oregon (Figure 1). The vineyards are planted in a variety of soils and experience various environmental conditions (Table 1). Geographical differences aside, these sites are all planted with the same clone of Pinot noir, farmed to produce super-premium wines, and the subsequent fermentations conducted with a high degree of winemaking replicability and control. All fermentations were conducted using the same set of process conditions to better observe site-specific differences.

In this study, it is hypothesized that the differences in fermentative behavior of vineyard sites can be quantified through the characterization of the yeast activity in real-time and changes in the chemical composition of the wine. By monitoring redox potential as a function of time during fermentation, the objective is to gain insights into the underlying biological and chemical differences between (1) vineyard sites and (2) fermentation replicates. From a process control standpoint, the temperature is controlled via water in the jacket of the fermentor along with pump activity for mixing [5]. Yeast activity increases temperature while decreasing the ORP, while air introduced from sampling and pump activity will increase ORP. Thus, we expect the fastest fermentations will have the most cumulative pump activity, necessary to maintain the temperature set point. Furthermore, we expect to see the extraction of color compounds increase during fermentation, correlating with increases in temperature and alcohol. We expect a high degree of reproducibility in terms of fermentation rate, ORP profile, and extraction between replicates but clear differences between vineyard sites. Analyzing the elemental profiles of each vineyard site will enable us to see how these sites differ across multiple vintages and may inform how elemental profiles correlate with fermentative performance and ORP profile. The combination of these data, typically not used to characterize wine fermentations, will help to elucidate previously “hidden” contributions of vineyard sites to fermentation outcomes and wine chemistry.

## 2. Results

### 2.1. Grapes from Different Vineyard Sites Result in “Slower” or “Faster” Fermentations

**Figure 2:**
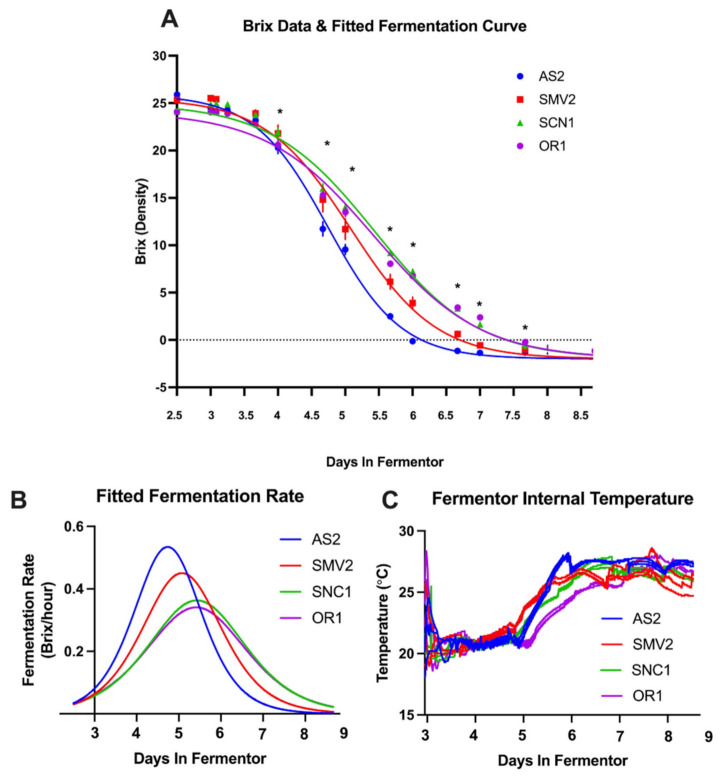
(**A**) The sigmoidal fitted curve reflects the decrease in density (Brix) proportional to the sugars consumed by the yeast; symbols denote manual sample points. A Brix value of 0 or below signals the end of alcoholic (primary) fermentation. The asterisk (*) between day 4 and 7.5 indicate where fermentations are significantly different from each other (as determined by Fisher’s least significant difference test, shown in Table S1, Supplementary Materials). (**B**) The fitted rate of fermentation plot shows the two faster fermentations (AS2/SMV2) display a higher initial rate of fermentation (i.e., rate of sugar consumption, Brix/h) than the two slower fermentations (SNC1/OR1). (**C**) The fermentor temperature of each replicate from inoculation through the end of fermentation (additional temperature data are reported in Supplementary Materials, Figure S1).

All of the vineyard sites, listed in Table 2, displayed typical fermentation profiles (shown in Figure 2A) and resulted in the yeast successfully converting sugars into alcohol within approximately 10 days (an acceptable period of time for winemaking) [2]. A typical fermentation curve is defined by an initial lag period, followed by an exponential consumption of hexoses (as measured by Brix), and a slowing of fermentation rate as the Brix measurement approaches negative values [1]. The fastest fermentation observed was with must from the AS2 vineyard site, which reached a fermentation rate of more than 0.5 Brix/h (i.e., rate of sugar consumption) and completed fermentation (negative Brix) by 72 h post-inoculation (Figure 2A,B). The SMV2 site achieved a maximum fermentation rate of more than 0.4 Brix/h, completed in 96 h. The musts from sites resulting in the slowest fermentations, OR1 and SNC1, were characterized by maximum fermentation rates of only 0.3 Brix/h and completed fermentation by 112 h (Figure 2A,B), which is a >50% increase in the overall fermentation time relative to AS2. While the absolute differences in fermentation times are minimal in these small volumes with well-controlled temperature, these differences would likely be more pronounced if these fermentations were conducted at a commercial production scale (>4000 L).

All of the fermentations began with similar fermentation rates when extrapolating back to inoculation at t = 0 (Figure 2B). By 40 h post-inoculation, the yeast in these fermentations had entered an exponential phase, during which they were actively growing, depleting oxygen, and consuming sugars [2]. The yeast population in the AS2 musts was extremely active between 24 and 64 h (Figure 2B). This increase in metabolic activity, as observed by the rate of change in density (by Brix), results in AS2 finishing fermentation fastest. The SMV2 fermentations also display a higher rate of fermentation early but reach a lower maximum rate than the AS2 fermentations. The OR1 and SNC1 fermentation rates remain relatively low throughout fermentation (Figure 2B). At the end of 9 days, the fermentations were considered dry by Brix, but enzymatic testing at press revealed that some replicates still contained residual sugar (RS) above 2 g/L, for instance, SMV2-Rep 1 (Table 3).

The temperature was closely controlled in each fermentation vessel because this parameter can greatly affect yeast activity and fermentation outcomes. After destemming into each fermentor, the must was cooled to ~7 °C and kept in “cold soak” for 3 days. Before inoculation, the fermentors were actively heated to 21 °C then maintained at this temperature until 48 h post-inoculation, when the temperature was allowed to passively rise to 27 °C (Supplementary Materials, Figure S1).

The two fastest fermentations had the most metabolically active yeast populations (as determined by maximum fermentation rate at 48 h). The AS2 fermentations displayed a steep increase in temperature between days 2 and 3 (Figure 2C); this increase is correlated with the rapid rate of fermentation and sugar consumption by yeast in the AS2 replicates (Figure 2A,B). When the temperature was allowed to rise from 21 °C to 27 °C, AS2 and SMV2 showed the fastest increase in temperature due to a yeast population rapidly producing heat while metabolizing sugars (as reflected in the fermentation rate).

### 2.2. Composite Redox Potential during Fermentation

**Figure 3:**
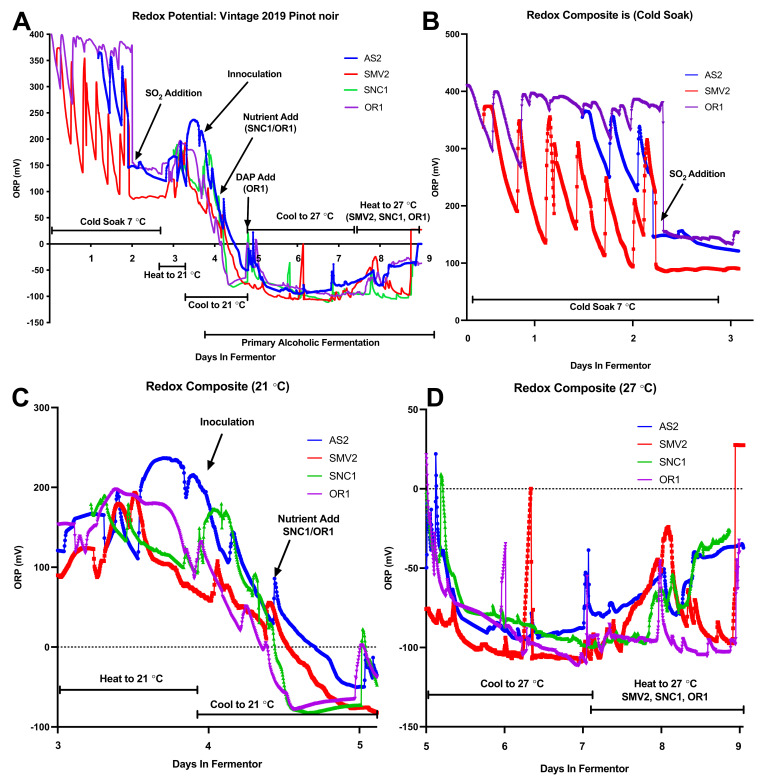
Each data point represents a 5 min interval. (**A**) Composite graph with the mean of all ORP measurements for each set of vineyard replicates over the entire time in fermentor. Process decisions are annotated, including changes to the temperature and nutrient additions. (**B**) The mean ORP measurements acquired during the initial 3-day cold soak period. The addition of SO_2_ caused an immediate decrease in the ORP value. (**C**). The mean ORP measurements acquired during initial heating, inoculation, nutrient addition, and the first ~24 h of fermentation. (**D**) The mean ORP measurements acquired during the middle and end of fermentation, including the period when temperature control was switched from cooling to heating (for selected conditions).

The Oxidation Reduction Potential (ORP) measurements are presented here as the mean redox value across replicates (Figure 3A). Probes were in place starting from when the must (mixture of skins and juice) was placed into fermentors to cold soak (3 days prior to inoculation) to the end of fermentation (when the solids were separated from the liquid). The probes captured the entirety of both cold soak and fermentation for SMV2 and OR1 replicates but missed the pre-inoculation period for AS2 and SNC1. As part of standard winemaking practices, the mixture of juice, skins, and seeds, otherwise known as must, was homogenized via “pump over” (automated mixing) for ~15 min twice per day. Pump overs assist with extraction, manage temperature gradients, and ensure accurate measures of Brix. The twice-daily pump-overs during fermentation coincide with manual sampling, which correlates with small increases in ORP every ~8–14 h throughout the cold-soak and fermentation (Figure 3A).

During the cold soak period (days 0–3) the ORP of the SMV2 started at ~350 mV then decreased considerably to ~200–100 mV between pump-overs, indicative of exogenous enzyme activity that consume molecular oxygen (e.g., polyphenol oxidase, laccase) and/or microbial metabolism (Figure 3B). The initial temperature of the SMV2 musts was also higher during cold soak (Supplementary Materials, Figure S1), which may have prompted microbial activity. OR1 displayed a lower magnitude of change with readings from ~400 to ~250 mV in the ORP between pump-over cycles. These differences in ORP values and patterns between sites are suggestive of must differences that could encompass different redox buffering capacities of the must, as well as differences in enzymatic or microbial metabolic activities. Near the end of day 2, ~40 mg/L of highly reductive SO_2_ (added as a solution of potassium metabisulfite—KMBS) was added to the fermentors, which caused a consistent drop (>250 mV) in the redox potential for all musts (Figure 3B).

On the morning of day 3, when the fermentors were actively heated to 21 °C, an increase in the ORP was observed, likely due to the mixing action of the pumps, which can introduce air and homogenize the temperature of the must (Figure 3B). Once the fermentors reached 21 °C, rehydrated RC212 (Lalvin) commercial yeast was inoculated (25 g/hL) into each fermentor, which was then pumped over for ~20 min to disperse the yeast (Figure 3C). The addition of inoculum and mixing resulted in an observable increase in ORP across all sites. By 12–24 h post-inoculation, the ORP declined to roughly −100 mV for all fermentations. This change in ORP is indicative of yeast metabolic activity, including the uptake of oxygen and excretion of reductive metabolites (Figure 3C) [40]. For SNC1 and OR1 ~12 h after inoculation, a nutrient addition was made to adjust the YAN, so these fermentations had a total of 250 mg/L (Figure 3C). This addition, which stimulates yeast metabolism, corresponded with a rapid drop in the ORP of these sites to the −100 mV level. Between days 4 and 5, the fermentations approach their lowest ORP minima (Figure 3C). Fermentations reached peak activity on day 4, with the yeast populations generating enough heat to require mixing to keep the temperature maintained at ~21 °C. This pump activity may have led to the spike in ORP on day 5 for AS2, SNC1, and OR1 (Figure 3D). The ORP declines as the temperature set point is changed from 21 °C to 27 °C. Across all conditions, ORP decreases, reaching an “ORP minimum” between days 6 and 7 (depending on fermentation kinetics), which coincides with the transition into the stationary phase. Near the end of fermentation (days 8–9), SMV2, SNC1, and OR1 were heated and mixed, corresponding to an increase in ORP values. The ORP for the finished AS2 and SNC1 fermentations is elevated (~50 mV) in comparison with SMV2 and OR1, which are still actively fermenting (Figure 3A,D).

### 2.3. Individual Redox Potential Profiles and Pump Activity

The Brix and temperature profiles, shown in Figure 2, are very similar within fermentation replicates (Brix Range of StdDev: 0.14–0.41). Tracking these traditional metrics of fermentation gives little insight into underlying differences between replicates. By inspecting the individual redox potential/ORP profiles, it is possible to identify differences between biological replicates that have previously not been quantifiable for wine fermentations. While the ORP values within each set of replicates follow similar trends, distinct points of divergence between sites and replicates occur as a result of the process control strategy. To control fermentation outcomes, the pumps ran automatically to maintain the temperature set point of each fermentor. By assessing the relationship between the observed ORP values and pump activity, we are able to infer the specific behavior of individual fermentations separate from the highly replicable Brix and temperature profiles.

**Figure 4:**
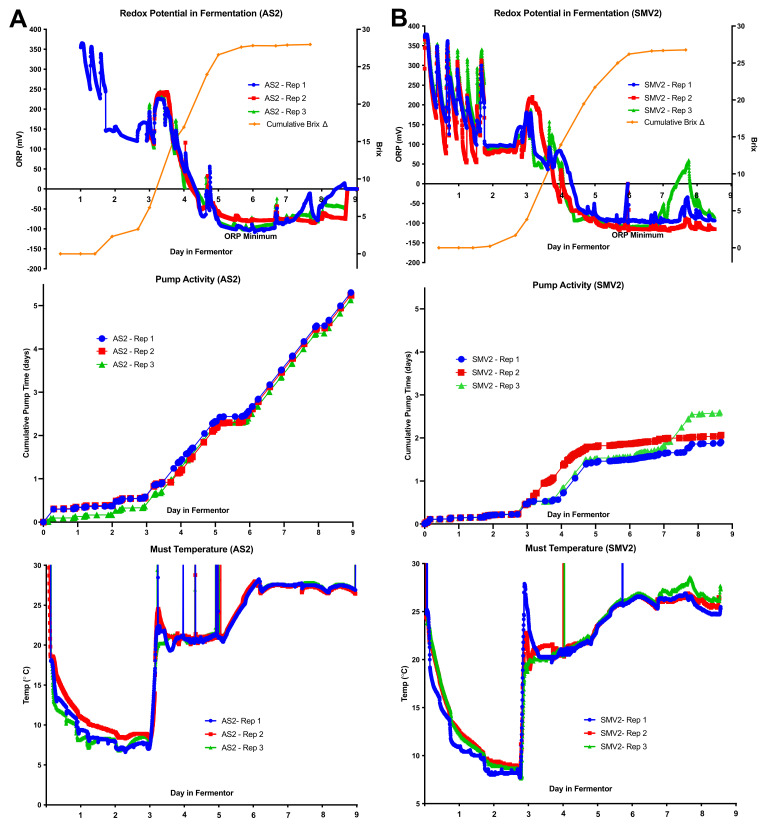

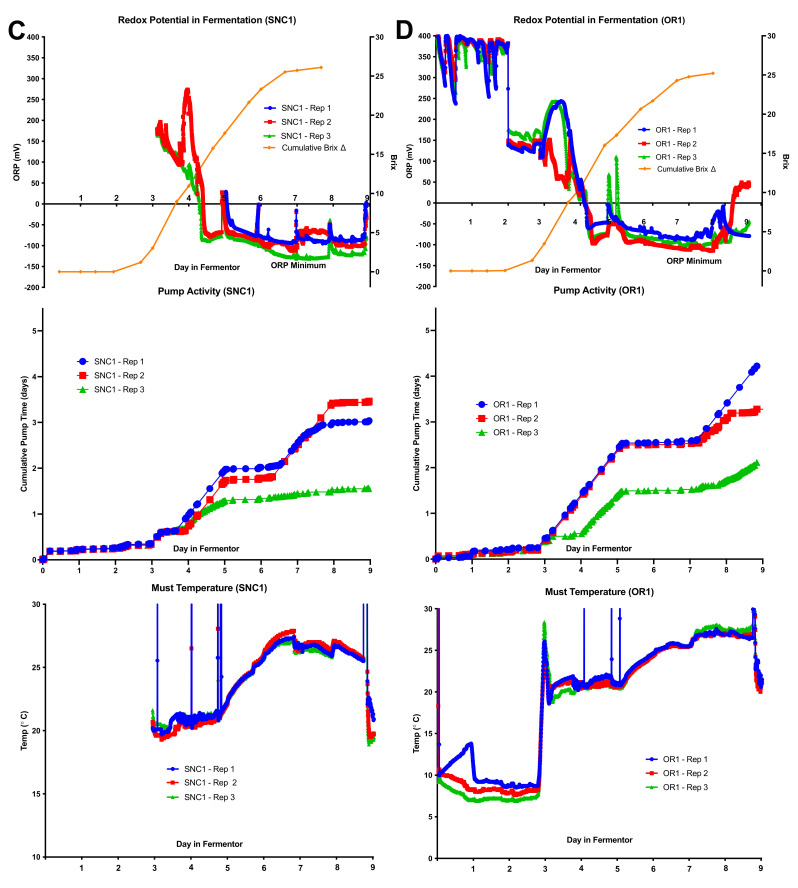
(**A**) Individual Redox Potential (ORP) profiles, cumulative pump activity, and must temperature profiles for the AS2 replicates. The AS2 replicates displayed the fastest and more reproducible fermentation profiles. (**B**) Individual Redox Potential (ORP) profiles, cumulative pump activity, and must temperature profiles for SMV2 replicates. SMV2-Rep 2/3 display a spike in ORP on day 6, corresponding with manual manipulation of these probes as they were lifted out of the fermentor for maintenance. (**C**) Individual Redox (ORP) profiles, cumulative pump activity, and must temperature profile for SNC1 replicates. SNC1-Rep 1/2 display a spike in ORP on day 6, corresponding with manual manipulation of these probes as they were lifted out of the fermentor for maintenance. (**D**) Individual Redox Potential (ORP) profiles, cumulative pump activity, and must temperature profile for the OR1 replicates. OR1-Rep 3 displays a spike in ORP around day 5 that corresponds with manual manipulation of the probe.

The AS2 replicates finish fermentation the fastest (by day 6), converting the majority of hexoses into ethanol between days 3 and 5 (Figure 4A). These replicate fermentations exhibit very similar trends in ORP, with the largest decrease in ORP corresponding to the maximum fermentation rate. During this period, the pumps for each fermentor were very active, mixing the fermentors to maintain the set temperature at 21 °C. Near the end of day 4, there is an abrupt increase in the ORP (~100 mV) due to almost constant pump activity maintaining 21 °C; once the temperature was adjusted to 27 °C, the pump activity ceased for ~1 day. After the abrupt increase in ORP, the ORP decreases to a minimum value of −100 mV on day 6, which is consistent with the metabolic shift from exponential to stationary phase (ORP Minimum). By days 7–8, the ORP increases, possibly due to air introduced from continuous mixing and because the yeast are less active at the end of fermentation, allowing the ORP to rise. The AS2 vineyard replicate fermentations were fairly consistent (Brix StdDev 0.22) and fermented the fastest. AS2 exhibited the highest cumulative pump activity (~5.5 days total) to maintain the temperature setpoints, corresponding with the fastest observed fermentations (Figure 4A).

The SMV2 replicates finish fermentation relatively quickly (between days 6 and 7), except for Rep 1 (see residual sugar in Table 3). The ORP values recorded during cold soak are indicative of enzymatic activity and/or microbial activity capable of consuming oxygen and decreasing the redox potential between pump-overs. This periodic fluctuation in ORP ceases after an SO_2_ addition on day 2. SMV2-Rep 2 required more mixing (~6 h) to reach the temperature set point, which is reflected by a high ORP value on day 3. With the ORP data, it is possible to observe how the yeast populations in the SMV2 replicates exhibited different behaviors and experienced different process conditions during the lag to the exponential phase transition, something which is not apparent from examining the Brix data. The variability in the activity of the yeast is specifically reflected in the divergent pump activity. Similar to AS2, the SMV2 fermentations decreased to the ORP minimum around day 6, consistent with the transition from exponential to stationary phase. On day 7, the process control was changed from cooling to 27 °C to heating to 27 °C. As a result, the ORP rises in a related manner for these replicates. SMV2-Rep 3 displays a proportional increase in pump activity (~150 mV) and ORP, while the ORP of SMV2-Rep 1/2 did not increase as much (~50 mV). The SMV2 replicates fermented quickly but required heating to maintain a temperature near the setpoint of 27 °C, and displayed the most variability in fermentation, with Rep 1 being “sluggish” (Brix StdDev 0.41). Overall, SMV2 fermentations stayed close to their temperature set points, displaying low cumulative pump run times (~2–2.5 days total).

The SNC1 replicates fermented more slowly than AS2 and SMV2 but faster than OR1 (finishing between days 7 and 8). SNC1 replicate fermentations received a nutrient addition on day 4 (Figure 4C). The ORP drops dramatically (~200 mV) immediately after the initial nutrient addition, possibly indicating that the nutrient composition, as well as subsequent stimulation of yeast metabolism, causes the ORP to decrease. Between days 4 and 5, the pump activity of SNC1-Rep 1/2 diverges from SNC1-Rep 3, indicative of differences in the heat produced by these fermentations. These replicates reach their ORP minimum around the end of day 6. On day 7, the temperature changed from cooling to 27 °C to heating to 27 °C, causing the ORP for SNC1-Rep 1/2 to rise but not for Rep 3. The regular action of the pumps for SNC1-Rep 1/2 days 7–8 can be visualized by an elevated (~50 mV) ORP value compared to SNC1-Rep 3. The SNC1 replicates fermented more slowly than AS2/SMV2 but were fairly consistent (Brix StdDev 0.12). The disparity in cumulative pump run time between replicates (~1.5 days vs. ~3–3.5 days) reveals that the yeast in SNC1-Rep 1/2 was more active during days 4–5 and days 7–8, thus requiring more temperature control during this period than SNC1-Rep 3 (which required less pump activity).

The OR1 replicates were the slowest to ferment of all sites. As with SMV2, the replicates showed some microbial/enzymatic activity during the cold soak, but with less magnitude of change in ORP during the same time period as SMV2 (~150 vs. ~300 mV), possibly due to the lower initial temperature of the must when compared with SMV2 (Figure 4B,D). As with SNC1, the nutrient addition on the morning of day 4 leads to an abrupt decrease in ORP (~150 mV). As the slowest fermentations, these replicates reach their minimum ORP between days 7 and 8. On day 7, the temperature changed from cooling to 27 °C to heating to 27 °C. This caused the ORP to rise then fall for OR1-Rep 1, to rise and stay elevated for OR1-Rep 2, and rise slightly for OR1-Rep 3, showing the inherent variability of replicate fermentations. OR1 replicates completed fermentation relatively slowly but were still fairly consistent (Brix 0.16 StdDev). The pump activity for OR1 displayed the biggest difference in cumulative pump run times (~2, ~3.25, ~4 days, respectively).

### 2.4. Anthocyanin and Iron-Reactive Phenolics

**Figure 5:**
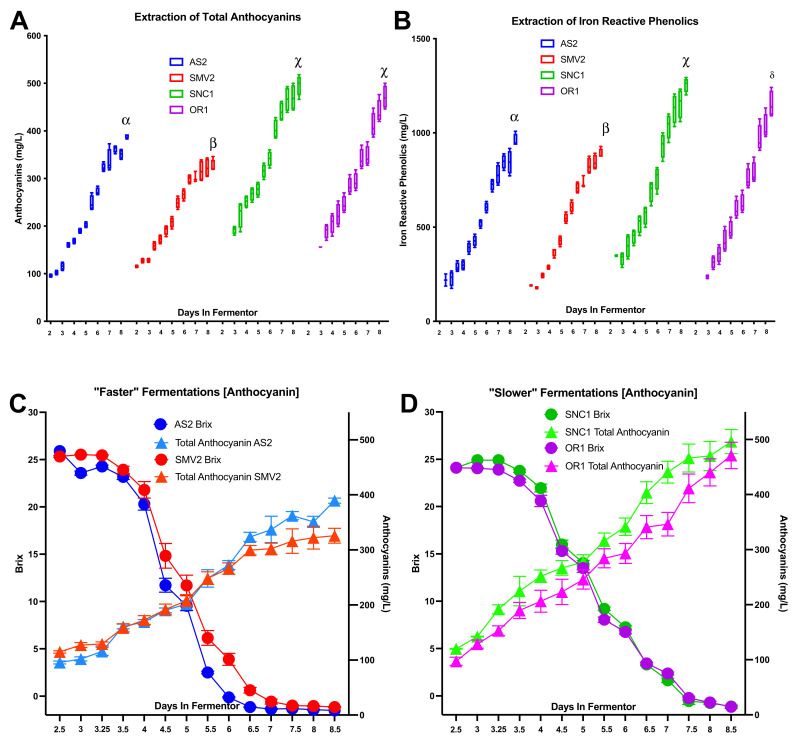
(**A**) Total anthocyanins over the course of fermentation, as measured by “Malvidin 3-glucoside equivalents”; Greek numerals denote significant differences in the values at the last time point as determined by Fisher’s LSD (least significant difference). (**B**) Total iron reactive phenolics over the course of fermentation, as measured by “Catechin equivalents”; Greek numerals denote significant differences in the values at the last time point as determined by Fisher’s LSD. (**C**,**D**) Visualization of fermentation kinetics between “faster” (**C**) vs. “slower” (**D**) fermentations and extraction of total anthocyanins (similar trends observed for iron reactive phenolics, data not shown). The “faster” site displayed a lower rate of extraction than “slower” sites, as reported in Figure 1B. Despite the “slower” fermentation kinetics, the amount of anthocyanins is relatively high for these conditions than in the “faster” conditions.

In conjunction with Brix measurements, anthocyanins and phenolics were assayed throughout fermentation by at-line UV-Vis spectroscopy to observe how the fermentation kinetics and ORP profile correlate with extraction. The initial measurements of anthocyanins showed higher concentrations in the SNC1 and OR1 musts than for AS2 and SMV2 (Figure 5A); this was also true for iron-reactive phenolics (Figure 5B). These trends continued throughout fermentation, with the SNC1 and OR1 sites finishing with a significantly higher concentration of color compounds than AS1 and SMV2, mean ratio of faster/slower total anthocyanins (0.74), and iron-reactive phenolics (0.77). The rate of anthocyanin extraction appears to correlate with the rate of fermentation (Figure 5C). AS2 and SMV2 had higher final ethanol concentrations than SNC1 and OR1, but this did not correlate with the highest concentration of phenolics or anthocyanins. The slower fermentations of SNC1/OR1 may have provided a more favorable matrix for the extraction of color compounds, with redox potential and temperature playing a factor in the observed differences [41].

### 2.5. Elemental Composition and Metal Ion Concentration

**Figure 6:**
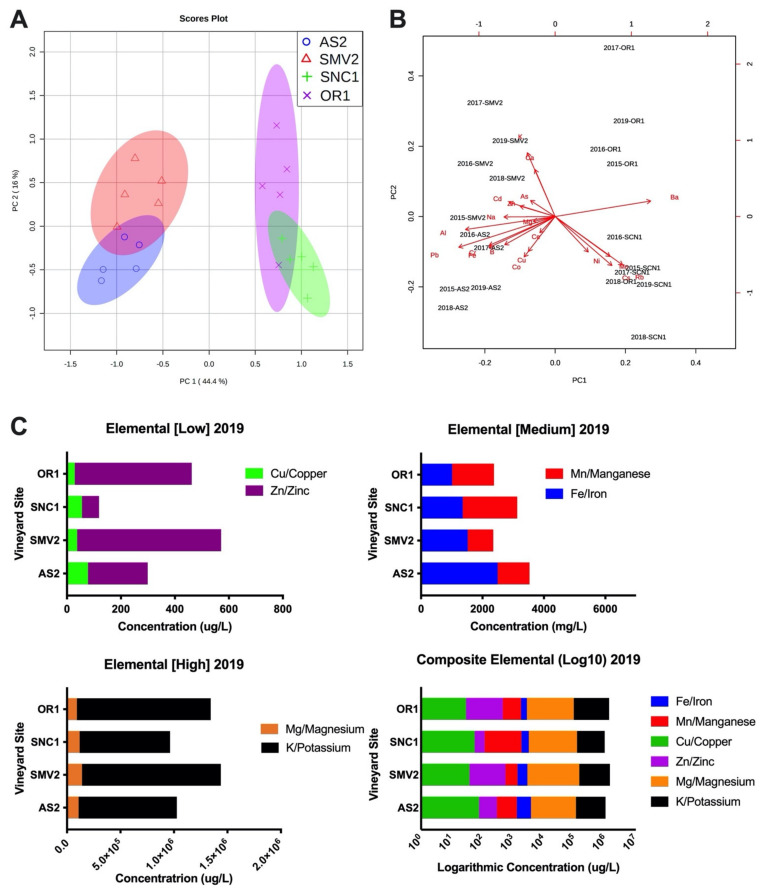
(**A**) Principal Component Analysis plot of elemental concentration and composition of the four vineyard sites; this analysis shows a clear segregation of the two “faster” and “slower” fermenting vineyard sites. PC1 captures 44.4% of the variation in the data set, while PC2 captures 16% of the variation in the data set. The ellipses represent a 95% confidence region. (**B**) As part of the same PCA analysis, the biplot shows the relative impact of which elements are driving the segregation of vineyard sites and vintages along PC1 (44.4%) and PC2 (16%). (**C**) Scaled concentration of yeast and redox relevant elements from the 2019 vintage; note the ratio of Fe/Mn between the faster (AS2/SMV2) sites vs. slower sites (SCN1/OR1). Check Supplementary Materials, Figure S3, for more Fe/Mn ratios.

In trying to understand the site-specific contributions to fermentation outcomes, the elemental profile of each wine was analyzed. The elemental composition is reflective of geography, soil type, and vineyard farming practices [33,42]. The individual vineyards sites separate robustly in two groups based on the concentration of elements observed across the five vintages (Figure 6A). Principal Component Analysis (PCA) shows the overlap of AS2 and SMV2 sites, which produce the “faster” fermenting musts, based on the SMV2 2015 vintage with the AS2 2016 and 2017 vintage (Figure 6A). By the same analysis, the sites SNC1 and OR1 that produce the two “slower” fermenting musts cluster, with the OR1 2018 vintage overlapping with SNC1 2015, 2017–2019 vintages. The most influential elements differentiating the two groups (OR1 and SNC1 vs. AS1 and SMV2) on the basis of the principal component (PC) axes are Ba, Pb, Al, and Fe along PC1 (Figure 6B). PC1 describes nearly 50% of the variation in these data, while PC 2 describes 16%. The differences driving the separation of AS2 vs. SMV2 and SNC1 vs. OR1 along PC2 are K, Ca (positive correlation) and Cs, Rb, Mn, Co (negative correlation) (Figure 6B). SMV2 and OR1 are positively correlated, while AS1 and SNC1 are negatively correlated with PC2. These data clearly show the segregation of “faster” and “slower” fermenting sites.

Individual elements in grape musts display a wide range of concentrations. The 2019 elemental profile data is best visualized as low- (Cu/Zn), medium- (Fe/Mn), and high- (Mg/K) concentration elements (Figure 6C). While individual elemental species vary by vintage, which is highlighted by the subtle variations in the relative positioning of sites across vintages in Figure 6B, the ratios of particular elements tend to trend similarly from year to year by site (Figure 6A). The most noticeable difference in elemental ratios between vineyard sites is the ratio of Fe/Mn. The sites (SMV2/AS2) that produce the two faster-fermenting musts have a higher ratio of Fe to Mn; this ratio is consistent across five vintages (Supplementary Materials, Figure S3), while the sites (OR1/SNC1) that produce the two slower-fermenting musts have a low ratio of Fe to Mn; this ratio is also consistent across five vintages (Supplementary Materials, Figure S3). The composition of biologically relevant elements could be predictive of yeast fermentation behavior and may influence the redox status of wine during aging [12,43]. The elemental profiles of the finished wines, especially with regard to the Fe:Mn ratio (Figure 4C), correlate strongly with fermentation outcomes in terms of rate and extraction.

## 3. Discussion

The goal of this work was to understand how site-specific factors affect fermentation outcomes by pioneering the utilization of combined real-time and at-line wine metrics. The vineyard sites for this study were chosen as part of a larger effort to characterize differences in Pinot noir grown in California and Oregon [33]. Pinot noir as a variety is considered to be particularly expressive of the geography, weather, soil type, microbes, and everything that contributes to the concept for “terroir”, resulting in differential must composition as a function of vineyard site [37,44,45,46]. The vineyard sites represent different microclimates/regions and soil types: AS2 (Arroyo Seco AVA) and SMV2 (Santa Maria Valley AVA) are on the Central Coast of California; SNC1 is on the Sonoma Coast (Sonoma Coast AVA), and OR1 is in Oregon (Willamette Valley AVA) (Figure 1) [33]. Based upon data from four previous vintages (2014–2018), the AS2 and SMV2 sites were selected as sites that produce “faster” fermenting musts, while SNC1 and OR1 were selected as sites that result in “slower” fermenting musts.

In comparing these vineyard sites, the climate is an important factor that affects the initial juice conditions. Site-specific climate conditions are relatively consistent from year to year based on data from previous vintages [33,42]. Given the geographic locations, sun exposure and weather are different for each site, yet the degree days for AS2, SMV2, and SNC1 are fairly similar (~1700–1900 degree days), except for the most northernly site OR1 (~1300 degree days). The total plant available water is similar for AS2, SMV2, and SNC1 (24.3–26.7 cm), but OR1 has soil characterized by much less available water (13.4 cm) [33,42] The highest Brix must came from AS2 (25.8), while the lowest Brix came from OR1 (23.1). This trend was also true for pH, as AS2 had an initial pH of 3.69, while OR1 had a pH of 3.43. The difference in growing conditions is also seen in the concentration of Yeast Available Nitrogen (YAN), which is much higher for AS2 (317 mg/L) than for OR1 (95 mg/L), which can affect not only fermentation kinetics but also the organoleptic properties of the wine [47]. To offset the initial difference in YAN, OR1 and SNC1 were amended with organic (NutriStart) and inorganic (DAP) nitrogen while in fermentors to reach a target YAN of 250 mg/L (across fermentations).

When attempting to understand yeast behavior in real-time, the metrics of Brix and temperature are insufficient to capture the biological and chemical nuances of fermentation. Brix is a temperature-dependent measure of density, which correlates with sugar in solution, but does not directly correspond with yeast metabolism [1]. The Brix curves for these wines exhibit small differences in kinetics between vineyard sites but do not reflect changes made to the process control strategy during fermentation (Figure 2). For these fermentations, the temperature was automatically controlled at every step of the winemaking process. Each set of replicates had four separate probes to measure temperature, mitigating technological bias (Supplementary Materials, Figure S2). The temperature was kept consistent through mixing by pump over, which introduced air, affecting the redox potential of the solution. The effect of pump activity was previously unknown, meaning ORP probes provided a novel metric to understand how changes in process control could impact fermentation outcomes [20].

By examining the ORP mean of replicates, it is possible to distinguish general trends in fermentation as they relate to important process decisions, for example, dosing with SO_2_, adding nutrients to stimulate yeast growth, and adjusting temperature set point (Figure 3A). Observing the individual ORP profiles reveals differences between replicates not visible in the Brix and Temperature data (Figure 4A–D). The observed ORP values provide insight into the growth of yeast, as the populations rapidly deplete oxygen during lag phase and decrease the ORP [48]. The oxygen is utilized as a nutrient during the lag and exponential phases to synthesize cell membrane components such as fatty acids and phospholipids [2]. The synthesis of these classes of compounds prepares the yeasts for successive rounds of division and helps to fortify the plasma membrane against proton stress and the dehydrating effects of alcohol [49]. Actively dividing yeast also decreases the redox potential by excreting reductive compounds (e.g., glutathione, protons) [21]. Previous work has shown that as a yeast population reaches an “ORP Minimum”, the biological and extracellular chemical production of H_2_S spikes, causing the yeast population to undergo a physiological transition from exponential growth phase into stationary phase [18,23]. Observation of the ORP profile can provide valuable insight into yeast population dynamics as well as how specific process decisions affecting fermentation progression.

The purpose of the cold soak period is to extract water-soluble pigments (anthocyanins) and enhance organoleptic properties such as mouthfeel [50]. The ORP values of SMV2 vs. OR1 during the cold soak demonstrate the difference in initial must conditions between sites (Figure 3B). In cold soak, the ORP of the must is most likely decreased by the metabolic activity of bacteria and non-*Saccharomyces* yeast but could also be impacted by enzymatic activity present in the must [51]. SO_2_ is added during cold soak to inhibit Polyphenol Oxidase activity, thus helping to maintain available oxygen for yeast and to mitigate browning reactions [52]. SO_2_ is also toxic/inhibitory to the bacteria and non-*Saccharomyces*; adding it helps to reduce microbial competition before inoculating with commercial yeast SO_2_ [1].

The AS2 vineyard site was the fruit harvested with the highest initial Brix and YAN (Table 1 and Table 2). The AS2 replicates fermented the fastest and were the most consistent (Brix 0.22 StdDev). From the perspective of process control, these fermentations produced enough heat to require almost constant cooling, with the highest cumulative pump activity (~5.5 days). The AS2 replicates were virtually identical, displaying consistent ORP and pump activity with very few points of divergence. At the end of 9 days in the fermentor, all replicates were “dry” (<2 g/L of sugar) (Table 3). These data are consistent with the hypothesis that the fastest fermentations will have the highest rate of sugar consumption and the most cumulative pump activity. From a winemaker’s perspective, these profiles represent an ideal set of fermentations; they finish alcoholic fermentation quickly and completely, freeing up fermentor space and minimizing the risk for oxidation or contamination.

The SMV2 vineyard site came in with the third-highest Brix and the second-highest YAN (Table 1 and Table 2). The SMV2 sites fermented second-fastest but also displayed more variability (Brix 0.41 StdDev) between replicates. The observed variability in the fermentation replicates can be understood by looking at differences in the ORP and pump activity, with deviation occurring around day 3 and again on day 7. This is most likely due to differences in the heating/mixing of the fermentors on those days in relation to changes in the temperature set point (Figure 2).

After 9 days in fermentors, the SMV2-Rep 2/3 replicates were finished with fermentation, but the SMV2-Rep 1 is incomplete, with an RS of ~17 g/L (Table 3). This vineyard site appears to disprove the hypothesis that the fastest fermentations have the most cumulative pump activity, speaking to the inherent variability of yeast despite nearly identical fermentation conditions. From a winemaker’s perspective, these fermentations were largely successful, but the incomplete replicate could be problematic in a commercial production environment, tying up valuable tank space and requiring special treatment.

The SNC1 vineyard site had the second-highest Brix but was third in YAN (Table 2). The SNC1 replicates were very consistent (Brix 0.12 StdDev) but fermented more slowly than AS2 or SMV2. As with SNC1, the Brix and temperature profiles are nearly identical, but the ORP and pump activity exhibit divergence between replicates (Figure 4C). The addition of organic nitrogen on day 4 caused a steep and almost immediate decrease in the ORP because the yeast was in an exponential growth phase; it readily uptakes the organic nitrogen (amino acids) and drops the redox potential [53]. The sudden increase in intracellular nitrogen facilitates the production and export of reductive compounds such as glutathione to help the yeast population mitigate environmental stressors (e.g., reactive-oxygen species) [54,55]. The set of fermentations agreed with the hypothesis that fermentations with less pump activity appear to correlate with lower rates of fermentation. For this set of replicates, SNC1-Rep 1/2 had cumulative run times of >3 days, while SNC1-Rep 3 required only ~1.5 days; it is likely that Rep 3 was close to, but did not exceed the temperature set point, thus not activating the pump, resulting in relatively low ORP values, especially between days 6 and 8 (Figure 4B). These fermentations completed with low residual sugar across these replicates (~4 g/L) (Table 3) demonstrate a similar rate of fermentation for all three replicates despite distinct differences in the pump over activity as a result of the temperature-based process control. From a winemaker’s perspective, the variability among these replicate fermentations could be standardized by changing the timing of nutrient additions and adjusting the temperature control and pump activity to achieve more uniform ORP values.

The OR1 vineyard site exhibited the lowest initial Brix and YAN compared to the other sites (Table 1 and Table 2). The pH of these grapes was also lower, which is consistent with the grapes grown in a cooler climate than other vineyard sites (Oregon vs. California) [33]. The OR1 replicates fermented the slowest of all the sites, possibly correlated with the low initial YAN (95 mg/L). On day 4, organic nutrients were added to adjust the YAN up ~250 mg/L. As with SNC1, these nutrients stimulated yeast growth/metabolism, causing a sharp decrease in the ORP due to reductive metabolites begin excreted. The effect of this stimulation is reflected in the pump activity for OR1-Rep 1/2 between days 3 and 5, but OR1-Rep 3 did not heat up as much and required the same duration of pump activity. The OR1 replicates displayed the slowest rate of fermentation, reaching the ORP minimum between days 7 and 8, later than any other site. The difference in pump activity between replicates is most likely due to Rep 3 being close to but not exceeding the temperature set point, therefore not activating the pump. The relative “sluggishness” of the OR1 replicates (compared to other sites) is reflected in the residual sugar >9 g/L at press. These data do not necessarily fit the hypothesis that pump activity is directly related to fermentation rate. From a winemaker’s perspective, these fermentations are not ideal, as slow fermentations monopolize tank space and can be more prone to oxidation and or spoilage. In a commercial production environment, these replicates could be managed through earlier additions of oxygen and micronutrients to increase the speed and efficacy of fermentation.

Through careful observation of this data, it is possible to qualitatively correlate changes in ORP with yeast activity during fermentation. These data demonstrate the value of utilizing redox potential as a real-time process parameter for winemaking. By measuring the ORP of these vineyard-specific fermentations, previously unobservable differences between biological replicates were revealed. Aberrations in the ORP profile between replicates provide insights into specific differences between the yeast and pump activity, as dictated by the temperature process control. The differences in pump activity can be most likely explained by the relatively small fermentor size used, with rates of heat generation closely matching the diffusion of heat for some of the replicates. Despite the observed differences in ORP, these experiments were extremely replicable (especially for wine fermentations). The Brix and temperature profiles were consistent within replicates (except for SMV2-Rep 3), while the ORP profiles all followed similar trends. The same set of experiments conducted in a winery without consistent process control would surely result in more variation between replicates.

ORP is a standard metric for other anaerobic fermentation industries (e.g., biofuel, dairy, water treatment) [7,8,9,10,56]. It is also an ideal process parameter for winemaking because it quickly reflects changes in yeast metabolism and the chemical environment of the fermentation [20]. Implementing ORP probes to monitor fermentations will give winemakers a nuanced understanding of how their decisions impact their yeast populations in real-time. Understanding the redox conditions during fermentation can help winemakers control the volatile aroma composition of their wines and potentially make predictions about longevity [20,22,57]. The redox conditions during fermentation are driven primarily by the microbes/yeast that is present but can also be influenced by enzymatic activity (especially during cold soak). The Redox potential during fermentation can determine the reactivity and speciation of metals (Cu/Fe/S) and, along with the extraction of color compounds (anthocyanins/polyphenols), can have a profound impact on the organoleptic properties of a finished wine [58,59].

The winemaking strategy employed for these vineyard lots was designed to mimic industry practices for Pinot noir. The two slower fermenting sites, SNC1 and OR1, have greater concentrations of color compounds than the AS2 and SMV2 sites (Figure 5). All vineyards are planted with the same clone of Pinot noir, so the observed differences are most likely due to site-specific factors such as sun exposure, vine orientation, and soil composition (Table 1) [33]. OR1 and SNC1 were from cooler climates and had significantly more anthocyanins and polyphenols extracted than either AS2 or SMV2 (Figure 5). Previous work has demonstrated that lower growing temperatures can result in high concentrations of color compounds [60]. The cold soak enables the extraction of anthocyanins to start, while the warm fermentation and the increasing concentration of ethanol help to extract polyphenols [5]. Two sites, AS2/SMV2, produced faster fermentations and finished with higher ethanol (14.1/13.9% ABV) in contrast to the sites SNC1/OR1, which produced slower fermentations and lower ethanol (13.3/12.7% ABV). The higher concentration of ethanol did not result in more extraction of polyphenols, which suggests that vineyard-specific conditions had more of an influence on the concentration of color compounds and their ability to be extracted than alcohol content alone. While qualitative correlations between ORP and phenolics appear from these data, future work is required to elucidate the effects, if any, of the relationship between extraction and ORP.

Another key component of analyzing site-specific variation is the elemental composition of the wines produced from each vineyard [32]. The elemental composition of wine is expressive of the regional microclimate, soil composition, farming practices, and vine physiology [33,42]. The elemental composition of these vineyards is relatively consistent from vintage to vintage (Figure 6a). The PCA of elemental composition by vineyard shows that the clear separation of sites that produce “faster” and “slower” fermenting musts is consistent over multiple vintages. However, slight variation between vintages highlights the impact of vintage growing conditions (e.g., water availability) as a likely factor in determining the concentration of particular elements every year [33]. The full relationship between fermentation kinetics, ORP, and elemental composition remains to be elucidated, but certain biologically active metals play an important role in yeast health during fermentation [43].

In a concentration-dependent manner, some of these elements can affect the kinetics of sugar consumption and yeast physiology [56]. Specifically, magnesium (Mg), zinc (Zn), calcium (Ca), potassium (K), manganese (Mn), copper (Cu), and iron (Fe) are all key nutrients for yeast during fermentation [34,61]. Too much or too little of these elements can negatively impact yeast health during fermentation [43]. The measured concentrations of these elements for the 2019 vintage are within or slightly below the recommended range for optimized yeast health [62]. Due to lower YAN, both the SNC1 and OR1 fermentations were supplemented with complex organic nutrients, containing an assortment of micronutrients which aim to correct any potential deficiencies. Although nutrient additions were made in a manner consistent with the manufacturer’s recommendations and industry norm, the observed differences in fermentation kinetics suggest that nutrient additions could be made earlier in fermentation to better compare site-to-site variation without initial nutrients being a possible determinant of performance.

From a biological perspective, Zn, Mn, and Cu are all relevant as enzymatic cofactors, and concentrations of these ions can strongly influence fermentative behavior [63]. From a wine stability and redox (ORP) perspective, Cu, Mn, and Fe are of particular interest, as the oxidation state of these metal ions determines their reactivity within the must/wine matrix [64]. From a chemical standpoint, the concentration and speciation of metals determine the impact that oxygen can have on the chemistry of the wine matrix [14,64]. The concentration, speciation, and association of these elements change as a function of the redox potential in fermentation as their oxidation state determines their reactivity in a solution [12,16]. Intracellular concentrations of speciated metals are tightly regulated, tied to cytosolic pH and intracellular redox conditions [65]. While there was not one particular element that appeared to drive the differences in fermentation kinetics, there is a robust correlation between the “faster” and “slower” musts with the ratio of Fe:Mn over multiple vintages (Supplementary Materials, Figure S3). Both Mn and Fe serve as important cofactors for key metabolic enzymes [1]. While it is unclear how, or if, this ratio of metals directly affects the dynamics of fermentation, it is strongly correlated with particular vineyard sites. Historically, wines from the OR1 vineyard site in particular exhibit relatively low iron concentrations and relatively slow fermentation kinetics. Through future work, we hope to elucidate connections between transition metal content, fermentation kinetics/ORP, and organoleptic traits as a function of wine matrix composition.

## 4. Materials and Methods

### 4.1. Vineyard Sites and Viticultural Practices

Grape clusters produced by *Vitis vinifera* L. cv. Pinot noir clone Dijon 667 were obtained from four different vineyard sites. The sites represent four different American Viticultural Areas (AVAs), which include Arroyo Seco (AR), Santa Maria Valley (SMV), Sonoma Coast (SNC), and Willamette Valley (OR). The distance between the southernmost and northernmost sites is approximately 1400 km, shown in Table 1. The site elevations span from ~300 to 600 ft, reported in Table 1. While there are some distinct differences between vineyard sites in terms of soil, topography, vine spacing, and row orientation, these sites are all farmed using similar viticultural practices (Table 1).

### 4.2. Fermentation and Inoculation Protocol

Grapes from the four different vineyard sites were harvested at 24.65 ± 1.14 Brix. Harvest dates ranged from 13 September through 30 September (Table 2). The mean pH was 3.6 ± 0.1, and the mean titratable acidity was 4.9 ± 0.5 g/L as tartaric acid (Table 2). The grapes were destemmed (but not crushed) into 200 L stainless steel fermentors, in quadruplicate, at the UC Davis Teaching and Research Winery (University of California, Davis, CA, USA).

A standardized winemaking protocol was used to minimize variation, with temperature functioning as the main process control parameter. The must was initially cooled to 7 °C and soaked at this temperature for 3 days. At the end of the second day, a 40 mg/L SO_2_ addition was made with a 15% (*w*/*v*) solution of potassium metabisulfite (KMBS). At the beginning of the fourth day, the must was warmed up to 21 °C in preparation for inoculation. On the afternoon of the fourth day, freeze-dried yeast (RC212 Lalvin, Lallemand, Petaluma, CA, USA) was rehydrated according to the manufacturer’s instructions and pitched at rate of 25 g/hL. For 2 days after inoculation, fermentation temperature was held at 21 °C, then allowed to passively rise to 27 °C. On the seventh day, the SMV2, SNC1, and OR1 ferments were switched from cooling to heating to 27 °C. On the ninth day after destemming, all lots were pressed off.

At inoculation, organic yeast nutrients (NutriStart, Laffort, Petaluma, CA, USA) were added to SNC1 and OR1 to supplement the Yeast Available Nitrogen (YAN) (Table 2). On the fifth day, nitrogen in the form of diammonium phosphate (DAP) (Omnisal GmBH, Lutherstadt Wittenberg, Germany) was added to SNC1 and OR1 (Table 2). Additional fermentation process details and temperature profiles are available in Supplementary Materials, Figure S1.

### 4.3. Initial Must and Final Wine Chemistry Analysis

Standard juice and wine chemistry characterization was performed at the UC Davis Teaching and Research Winery. Standard analyses include pH, titratable acidity (TA), malic acid, ammonium (NH_3_), Primary Amino Nitrogen (NOPA), residual sugar (RS), acetic acid, free SO_2_ (FSO_2_), and *v*/*v*% ethanol (Table 2). Wines were centrifuged to remove solids and sparged with N_2_ gas to remove CO_2_. Ethanol concentrations (*v*/*v*%) were determined by an Anton Paar Wine Alcolyzer (Anton Paar USA Inc., Ashland, VA, USA); a 14 *v*/*v*% model wine solution was used as a single point calibration. pH was measured with a Thermo Scientific ORION 5 STAR benchtop (Thermo Fisher Scientific, Waltham, MA, USA). A two-point calibration at pH 3 and pH 4 was used. Titratable acidity, free SO_2_ was measured with a Mettler-Toledo DL50 titrator (Mettler-Toledo Inc., Columbus, OH, USA). Residual sugar was measured using Thermo Scientific Gallery analyzer (Thermo Scientific, Waltham, MA, USA) [25]. This analysis was accomplished using a standard acid–base titration with 0.1 N NaOH; the output is reported in tartaric acid equivalence.

### 4.4. Process Control and Probe Installation

Fermentation conditions, temperature set points, and data collection were controlled by Integrated Fermentation Control System (IFCS) units (Cypress Semiconductor, San Jose, CA, USA) [5].

Platinum electrode 120 mm Arc ORP probes (Hamilton Company, Reno, NV, USA) were connected to a 120 Ω terminated RS-485 bus, and a Modbus gateway (Stride, SGW-MB1511-T) was used to sample and store probe data into internal memory. By default, the ORP probes have a Modbus address of 1 and use a 19,200 baud rate, with eight data bits, one stop bit, and no parity bit. The Modbus address of each probe was programmed to be unique to avoid conflicts on the bus. A time-series database (PI, OSIsoft, San Leandro, CA, USA) was used to read the gateway’s internal memory through a Modbus TCP/IP interface. The ORP, probe temperature, and probe resistance were recorded as a function of time.

Custom 3D-printed enclosures were designed and fabricated out of food-grade filament (TRUE Food Safe PETG) (Filaments.CA, Mississauga, ON, Canada) to protect ORP probes from damage (photos in Supplementary Materials, Figure S2). Parafilm was wrapped around the connections and ensure a watertight seal. The enclosed probe was placed in a stainless-steel screen at the center of the fermentors. Hollow, polypropylene balls (9338K13, McMaster Carr, Aurora, OH, USA) were placed in the screen beneath the probe to allow the sensing platinum electrode to remain in contact with the top of the juice as the liquid level changed throughout fermentation.

### 4.5. Elemental Analysis of Must

The elemental composition of the four vineyard sites was characterized at the UC Davis Interdisciplinary Center for Plasma Mass Spectrometry, ICPMS.UCDavis.edu (University of California, Davis, CA, USA) by inductively coupled plasma mass spectrometry (ICP–MS) using an Agilent 8900 (Agilent Technologies, Palo Alto, CA, USA). The analytical method was based on that of Hopfer and colleagues [32] with minor modifications. Forty-seven elements were profiled in a mass range of 7–238 *m*/*z*. Literature has shown that these elements profiled have been detected in wines [32].

Wine samples were collected in 15 mL metal-free polypropylene centrifuge tubes (VWR International, Visalia, CA, USA) and stored at 4 °C prior to analysis. Wine samples were diluted 1:3 and 1:100 in 5% HNO_3_ (*v*:*v*, conc. Tr. Metals HNO_3_:18.2 MΩ/cm water) and 5% HNO_3_/5.5% MeOH, respectively, to enable a broad range of elements and concentrations to be quantified. Standards were prepared in 5% HNO_3_/5.5% MeOH to matrix-match the diluted wine samples. Commercially available mixes and individual elemental standards, vide supra, were purchased to create calibration curves. These calibration curves were prepared with 11 points between 0.5 and 5 × 10^4^ μg L^−1^, made daily from stock solution that included 47 elements.

Additional information on calibration, sample preparation, instrument, sample loading, settings, injection, and data processing can be found in [32,33].

### 4.6. Brix/Phenolic Sampling and Analysis by UV-Vis Spectroscopy

After destemming into each of the fermentors, the liquid was sampled 1–2 times daily, at approximately 10 a.m. and 4 p.m. local time (Davis, CA, USA). This sampling frequency excludes the first and ninth days after destemming, on which the fermentors were only sampled once. On the first day post-inoculation, each fermentor was sampled at approximately 4 p.m., and on the ninth day post-inoculation, they were sampled at approximately 10 a.m. The ninth-day sample was the final sample for each fermentation vessel prior to separating the liquid wine from the solid pomace by pressing. Samples for UV-Vis analysis were taken from the fermentation vessels via the vessel’s dedicated pump-over mechanism [32]. Each fermentor was pumped over for 2 min prior to a sample being removed. Approximately 50 mL of juice was collected from each fermentor. These samples were all placed in 50 mL conical centrifuge tubes. An Anton Parr DMA 35 Basic densitometer (Anton Paar USA Inc., Ashland, VA, USA) was used to take Brix measurements of each 50 mL sample removed from each fermentor.

After Brix measurements were recorded, 2 mL of juice was removed from each of the 50 mL samples and placed into a 2 mL centrifuge tube. The samples were then centrifuged (Fisher Scientific accuSpin Micro 17, Thermo Fisher Scientific, Waltham, MA, USA). The centrifuge was set to 13,300 rpm and was run for 5 min. Once the 2 mL samples had been centrifuged, they were decanted into a second set of 2 mL tubes. This second set of tubes was used to hold the samples to be characterized by UV-Vis spectroscopy (Thermo Scientific Genesys 150 Spectrophotometer, Thermo Fisher Scientific, Waltham, MA, USA). UV-Vis absorption spectra of samples were collected from 200 to 900 nm by using a quartz flow cell with a path length of 0.1 mm.

Samples were drawn into the spectrophotometer cell via a peristaltic pump (Cole-Parmer Masterflex L/S, city, state abbreviation if USA, country). Thermo Fisher VISIONlite software (Thermo Fisher Scientific, Waltham, MA, USA) was used to control the spectrophotometer and record the UV-Vis spectra. For each sample, the UV-Vis spectrophotometer scanned from 900 nm to 200 nm. The recorded spectra were uploaded to the WineXRay website (www.winexray.com, accessed on 3 February 2021), where the phenolic calibrations are performed. These calibrations produced the phenolics data reported in this paper.

### 4.7. Figure Generation, Data Processing, Statistics

All tables, figures, and statistics (with the exception of Figure 6A,B) were generated using the Prism 8 software (GraphPad, San Diego, CA, USA). For Figure 1, fitted fermentation curves were modeled using the Sigmoidal dose–response (variable slope) equation in Prism, fitting the Brix data to a logistic function with a minimum value of −2 and the max Brix value, solving for ×0 and L. For Figure 1 and Figure 5, OriginPro version 2021 (OriginLab Corporation, Northampton, MA, USA) was used to perform one-way Analysis of Variance (ANOVA) on the data to determine significance (*p* = 0.05); a Fisher’s least significance difference (LSD) test was subsequently used to determine significant differences in fermentation outcomes (Brix, anthocyanins, or iron reactive phenolics at various time points) of musts between vineyard sites. Figure 6A,B the PCA, and biplot were generated with elemental concentration data normalized by range scaling in MetaboAnalyst 4.0 [66].

## 5. Conclusions

With this work, site-specific factors and their influence on fermentation outcomes are explored, including qualitative relationships between fermentation kinetics, yeast activity, redox potential, phenolic extraction, and elemental composition. Compelling correlations that merit further investigation are reported. The utilization of ORP probes to track replicate fermentation demonstrates the effectiveness of this metric to better understand yeast activity and winemaking steps in real-time. This work highlights innovative ways for winemakers to follow the chemical and metabolic processes that would otherwise be “hidden”. ORP as a parameter is extremely sensitive to process changes, reflecting automatic and manual operations of fermentation conditions. Observable differences in the ORP between replicate fermentations and different sites appear to be strongly correlated with mixing/pump activity/sampling (air introduction). Extraction of color compounds is a function of site/origin that may be affected by fermentation kinetics and the redox conditions. Monitoring the phenolic extraction profile could provide another metric by which fermentation outcomes can be predicted or controlled by changing conditions in a directed manner. Analysis of the elemental composition revealed reproducible differences between vineyard sites; in particular, the ratio of Fe:Mn correlates with fermentation kinetics. For future vintages, we will continue to profile fermentations by tracking ORP, as well as experiment with controlling the fermentative environment via an ORP setpoint. Our work is directly applicable to industry partners who want to use ORP as a process parameter to help inform the timing of nutrient additions, including O_2_, and to control fermentation outcomes.

## Figures and Tables

**Table 1:** Vineyard information and viticultural details for on each site included in this study. Including American Vineyard Area, elevation, year of planting, soil type, topography, vine spacing, and row orientation.

Vineyard Site	American Vineyard Area	Elevation (ft)	Year of Planting	Soil Texture	Topography	Vine Spacing (ft)	Row Orientation
AS2	Arroyo Seco	591	2005	Loam	Hillside	4 × 3	E/W
SMV2	Santa Maria Valley	503	2004	Loam	Bench	6 × 5	NE/SW
SNC1	Sonoma Coast	667	2000	Sandy Loam	Hillside	9 × 5	NE/SW
OR1	Willamette Valley	309	2005	Clay Loam	Bench	3.28 × 5.25	N/S

**Table 2:** Initial must chemistry and composition. Data in this table include: harvest dates, Stem:Must ratio, pH, titratable acidity, malic acid, free ammonia, Primary Amino Nitrogen (NOPA), Yeast Available Nitrogen (YAN), as well as the amount of NutriStart, and Diammonium Phosphate (DAP) nutrients added to SNC1 and OR1.

Vineyard Site	Harvest Date	Initial Brix	Stem:Must Ratio	pH	Titratable Acidity (g/L)	Malic Acid (mg/L)	NH_3_ (mg/L)	NOPA (mg/L)	YAN (mg/L)	NutriStart (g)	DAP (g)
AS2	9/16/19	25.8	0.057	3.69	5.23	3772	127	255	317	0	0
SMV2	9/24/19	24.6	0.068	3.69	4.89	3105	90.3	186	258	0	0
SNC1	9/13/19	25.1	0.066	3.58	4.23	3067	98	133	211	36.1	1.3
OR1	9/30/19	23.1	0.049	3.43	5.35	4307	26	74	95	32.6	39.2

**Table 3:** Final wine chemistry of the blended fermentation replicates. Blends were made after malolactic fermentation by aggregating wine in the four replicate fermentation vessels. Residual sugar (RS) is an indication of fermentation “completeness”. RS is reported for each biological replicate across vineyards. Standard chemical measurements for finished wine are presented for each vineyard site as a mean of the blended fermentation replicates, along with the standard deviation for each set of technical replicates.

Vineyard Site	ALC %	pH	Titratable Acidity (g/L)	Acetic Acid (g/L)	Free SO_2_ (mg/L)	Rep 1 RS at Press (g/L)	Rep 2 RS at Press (g/L)	Rep 3 RS at Press (g/L)	RS in Bottle (g/L)
AS2	13.93 ± 0.01	3.75 ± 0.01	5.19 ± 0.05	0.42 ± 0.01	31.33 ± 0.03	0.96	0.84	1.05	0.60 ± 0.58
SMV2	14.09 ± 0.01	3.74 ± 0.00	5.35 ± 0.03	0.44 ± 0.02	31.67 ± 0.02	17.35	3.96	1.68	0.57 ± 0.58
SNC1	13.33 ± 0.01	3.53 ± 0.01	5.58 ± 0.02	0.25 ± 0.01	17.67 ± 0.03	4.06	3.25	4.74	0.55 ± 0.58
OR1	12.71 ± 0.01	3.66 ± 0.01	5.34 ± 0.05	0.33 ± 0.01	26.67 ± 0.02	9.29	10.4	10.78	0.23 ± 1.15

## Supplementary Materials

The following are available online: Figure S1: Temperature in fermentor, Figure S2: Probe/fermentor setup, Figure S3: Iron/manganese ratio by vintage, Table S1: Fermentation profiles of the musts from the four different vineyard sites at various sampling time points from 16 to 112 h.

## Sample Availability

Samples are not available from the authors.
