# Supplementary material for: Monitoring Site-Specific Fermentation Outcomes via Oxidation Reduction Potential and UV-Vis Spectroscopy to Characterize “Hidden” Parameters of Pinot Noir Wine Fermentations
Source: Molecules. 2021 Aug 5;26(16):4748. doi: 10.3390/molecules26164748 (PMC8400154; doi:10.3390/molecules26164748)
Supplement: Supplementary file 1 [file molecules-26-04748-s001.zip › molecules-1239994-supplementary.pdf]

Supplementary Materials

Monitoring Site-Specific Fermentation Outcomes via Oxidation Reduction Potential and UV-Vis Spectroscopy to Characterize "Hidden" Parameters of Pinot Noir Wine Fermentations

Gordon A. Walker [1], James Nelson [2], Thomas Halligan [3], Maisa M. M. Lima [1], Andre Knoesen [2] and Ron C. Runnebaum [1,3,*]

1 Department of Viticulture & Enology, University of California, Davis, CA 95616, USA; gawalker@ucdavis.edu (G.A.W.); mmlima@ucdavis.edu (M.M.M.L.)
2 Department of Electrical and Computer Engineering, University of California, Davis, CA 95616, USA; jjnel@ucdavis.edu (J.N.); aknoesen@ucdavis.edu (A.K.)
3 Department of Chemical Engineering, University of California, Davis, CA 95616, USA; twhalligan@ucdavis.edu
* Correspondence: rcrunnebaum@ucdavis.edu; Tel.: +15-30-752-9078

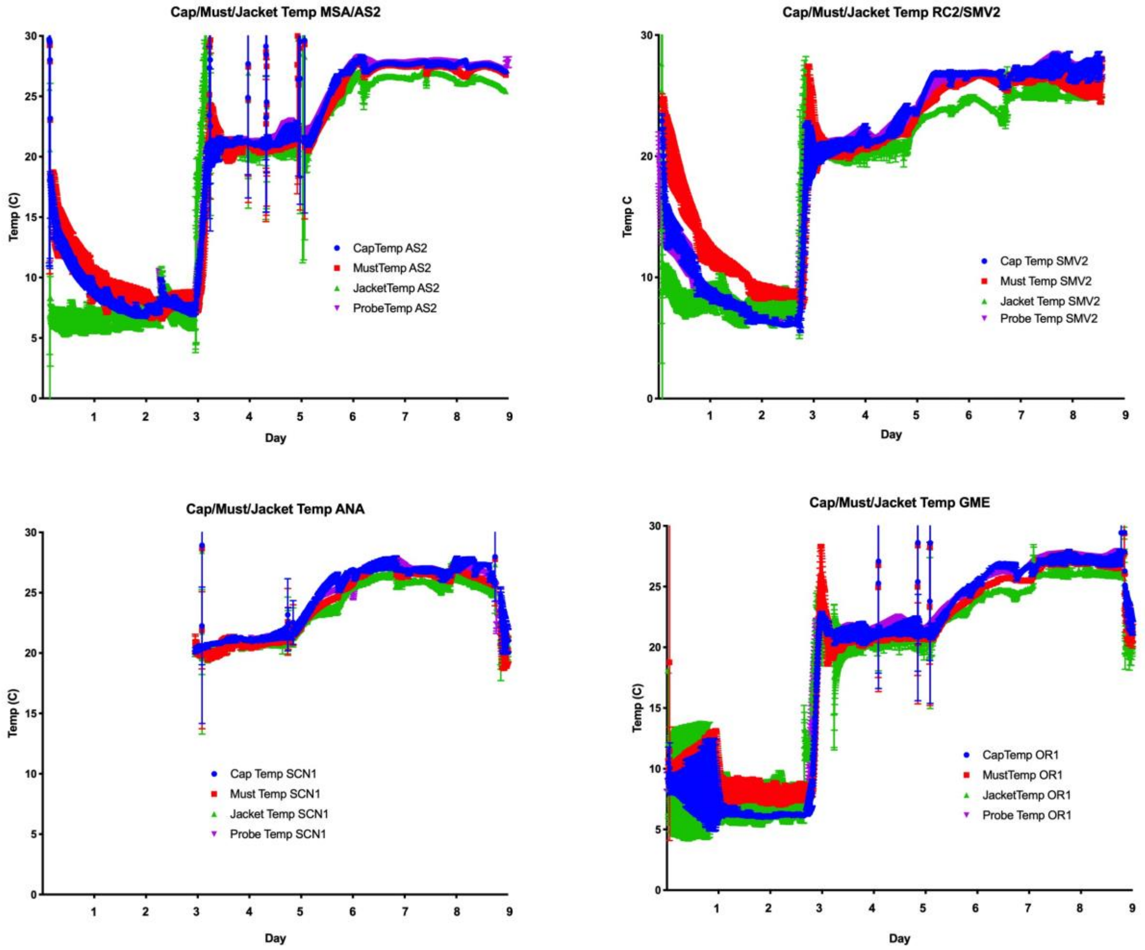

Figure S1. Temperature in fermentor. Temperatures for all vineyard sites and replicates are presented. Temperature was recorded with four different probes (Cap, Must, Jacket and ORP Probe) for each condition. The data shows small deviations based on the measurement control for each probe location, but shows similar similar trends across all conditions and replicates.

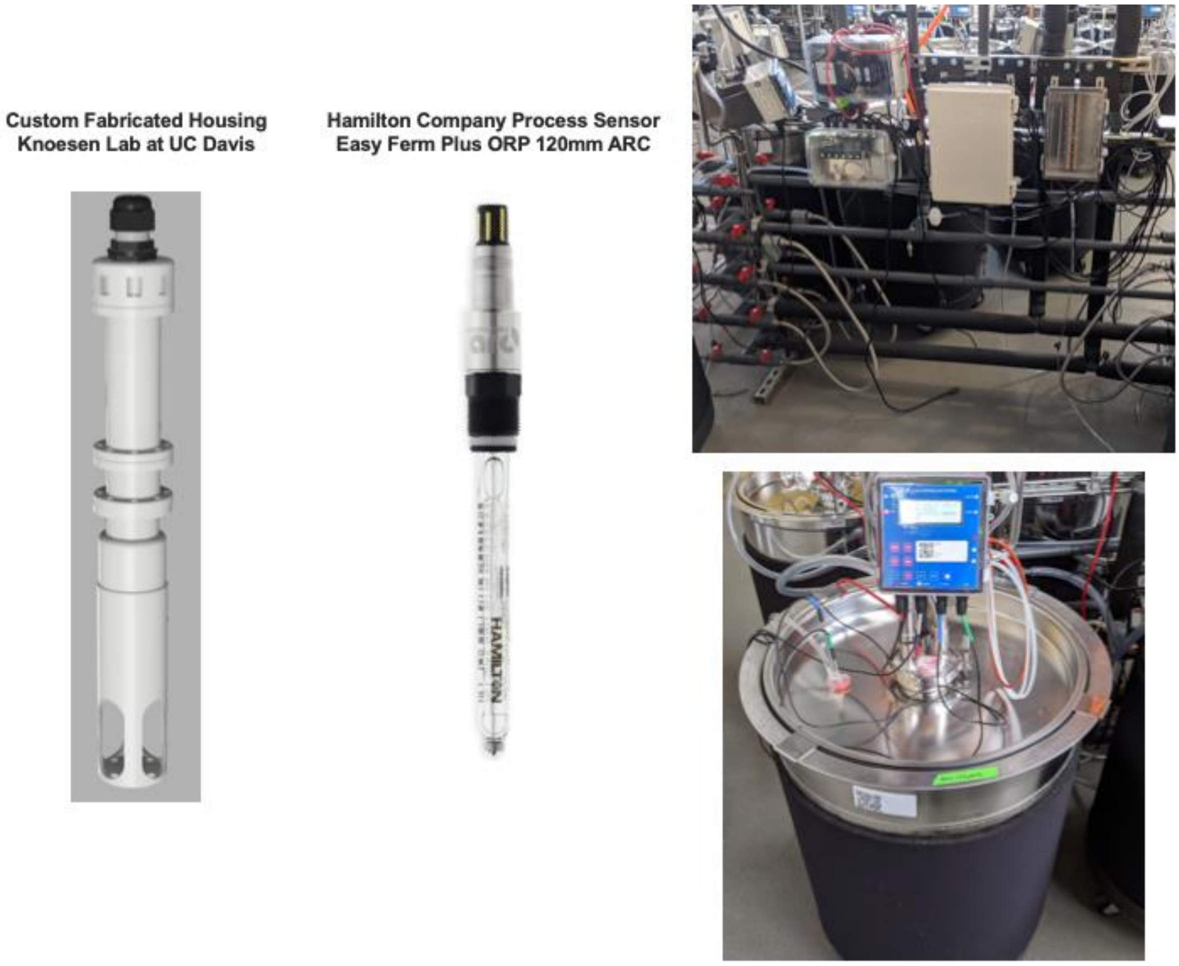

Figure S2. Probe/fermentor setup. A diagram of the custom 3D printed housing for the Hamilton ORP probes. Photos of the communication boxes and fermentor set up for ORP probe installation in the UC Davis Research Winery.

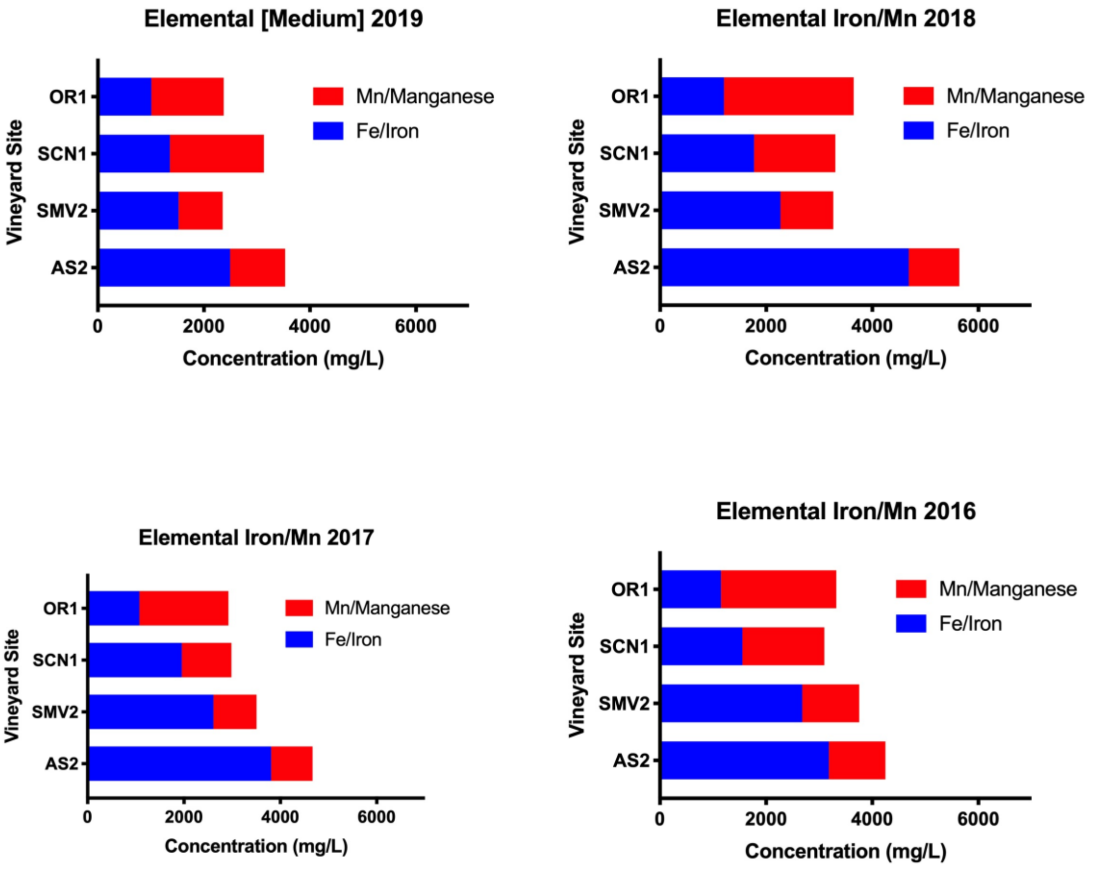

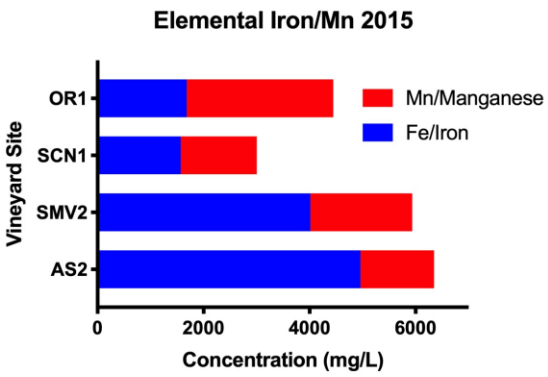

Vineyard	Average Ratio	Standard Deviation
AS2	3.67	1.03
SMV2	2.32	0.41
SNC1	1.19	0.43
OR1	0.59	0.10
Units	(Fe mg/L)/(Mn mg/L)	

Figure S3. Iron/manganese ratio by vintage. Iron to manganese ratios quantified over 5 vintages. There is variation within each vintage, but the ratio of Fe:Mn is fairly consistent, especially for the OR1 vineyard.

Table S1. Fermentation profiles of the musts from the four different vineyard sites at various sampling time points from 16–112 h. Data reported are average density measurements (Brix) of four replicate fermentations for each site.

	**16 h**	**26 h**	**40 h**	**50 h**	**64 h**	**74 h**	**88 h**	**98 h**	**112 h**
**Site**					**Brix**				
OR1	22.7 c	20.6 b	15.3 a	13.5 a	8.0 b	6.7 a	3.4 a	2.4 a	−0.2 a
SNC1	23.8 a	21.9 a	16.0 a	14.0 a	9.2 a	7.2 a	3.3 a	1.6 b	−0.5 a
SMV2	23.9 a	21.8 a	14.8 a	11.7 b	6.1 c	3.9 b	0.6 b	−0.5 c	−1.0 b
AS2	23.2 b	20.3 b	11.7 b	9.5 c	2.5 d	−0.1 c	−1.1 c	−1.3 d	−1.3 b
